# Supplementary material for: A scientometric analysis of birth cohorts in South Asia: Way forward for Pakistan
Source: PLoS One. 2020 Jul 9;15(7):e0235385. doi: 10.1371/journal.pone.0235385 (PMC7347181; doi:10.1371/journal.pone.0235385)
Supplement: Attachment — Submitted filename: Response to reviewers.docx [file pone.0235385.s002.docx]

Dear Dr. Man Ki Kwok,

My co-authors & I are very grateful to you for an excellent feedback on the manuscript. We have revised our manuscript in line with your suggestions and provide line by line responses below. With these revisions, the quality of the manuscript has improved substantially.

I believe the major change in the revised manuscript is its shift from a systematic mapping study design to a scientometric and bibliometric study design. These methods are very common in library sciences and knowledge mapping fields but a rare occurrence in healthcare sciences. PloSOne has recently published several studies in this domain, including our recent paper on online hate:

**Reference:** Waqas A, Salminen J, Jung SG, Almerekhi H, Jansen BJ. Mapping online hate: A scientometric analysis on research trends and hotspots in research on online hate. PloS one. 2019 Sep 26;14(9):e0222194.

We have now described these methods in more detail and tried to draft it in a more reader friendly manner. We have also avoided cognitive leaps from descriptive to inferences and provided justification for the design of these study. Please, find detailed responses to each comment below.

We are sure that this study would attract interdisciplinary leadership including scientists, policy makers and funders. It would also be a preamble to establishment of birth cohorts in Pakistan. We look forward to a favorable decision in due time.

Best wishes,

Dr. Ahmed Waqas

Corresponding author

**Editor comments**

**Comment 1**

Please fully address all helpful and useful comments from the reviewer, including more comprehensive and detailed searching, extraction, coding and analysis, clearly distinguish between descriptive summary and analytical findings, avoiding cognitive leap from descriptive to inferences, and justification on whether this is a narrative review, systematic review, meta-analysis, systematic map.

**Response**

Dear Dr. Man Ki Kwok, thank you for the excellent feedback. We believe these comments are very valid. We have revised our manuscript in line with these comments. This has raised the quality of the manuscript and improved the prospects of reproducibility. Please, find point to point responses below:

1) **Comprehensive & detailed searching**

For the purpose of this analysis, we only chose one database i.e. Web of Science (core databases). This is because unlike other databases such as scopus or pubmed; Web of Science has bibliographic details of indexed studies as well as the studies they are cited in. These bibliographic records thus, allow us to study co-citation relationships among different studies published in a domain. It follows the basic principle that if study A and B, both cite study C, they are said to have a co-citation relationship.

We have therefore, added following information to reflect this:

**Methods**

*Line 100-101: This study was conducted as per the principles of knowledge mapping and co-citation analyses outlined by Chen et al., [23,24].*

*Line 106-108:* *For the purpose of scientometric analyses, only Web of Science (core databases) were searched. This database records citing references of indexed studies, necessary for study of co-citation relationships in literature [23,24].*

**Comment 2**

**Extractio**n**, coding and analysis**

For this section, we have made the following changes:

a) In the methods section, we have clearly defined two phases of data analyses: bibliometric (manual data abstraction) and scientometric analyses (using citespace software).
b) *Out of 260 full texts, 38 studies were excluded for reasons including regions other than South Asia (n= 21), other study designs (n= 11), duplicate texts (n= 4) and literature reviews or systematic reviews (n= 2). (Line 132 to 134).*
c) For data abstraction procedures for bibliometric analyses, following information is added:

*“Then, data pertaining to characteristics of these studies were manually extracted from abstracts and entered in Microsoft Excel sheets. The data abstraction phase was performed by one reviewer (AW) who noted study characteristics including country, city/state, journal and year of publication, major research areas, funding and institutional affiliation. Moreover, during this data extraction exercise, unique cohorts were identified. Thereafter, several additional variables such as sample size, study design, primary hypotheses, time period, location and variable measurements were extracted for this subset of cohorts”.*

**Comment 3**

**Clearly distinguish between descriptive summary and analytical findings, avoiding cognitive leap from descriptive to inferences**

a) To distinguish between descriptive summary and analytical findings, we have restructured the results and discussion section. Results have now been divided into sections: i) **Descriptive and bibliometric analyses ii)** **Scientometric & co-citation analyses**

This should distinguish between results obtained using manual abstraction of data and those using citespace software.

b) The section ***Descriptive and bibliometric analyses*** following subsections: Research output, Major birth cohorts in Pakistan and Summary of pervious cohorts in South Asia.
c) The section ***Scientometric & co-citation analyses*** has the following subsections: Top organizations, funding bodies and journals, Top keywords of Birth Cohort Research in South Asia, Foci of Research & important studies from Birth Cohort Research in South Asia.
d) We have also removed any points int his section that would better fit in discussion section. For instance, the following paragraph in results section has been deleted:

*Co-citation analyses of the limited research contributions from birth cohort studies in South Asia reveal that these are clustered into a few specific clusters pertaining to physical health. And in contrast to the British cohorts, there has been negligible contributions in the area of psychological health of mothers and children. Moreover, the recent holistic drive in global health in delineating the psychoscocial determinants of diseases- thus, defining physical and psychiatric morbidities using both the biological and psychosocial approaches, is unseen in the region.*

**Comment 4**

**Justification on whether this is a narrative review, systematic review, meta-analysis, systematic map**

**Response**

We have now removed any mention of systematic mapping from the manuscript. The design of the study has now been correctly defined as scientometric analyses.

**Comment 5**

In addition, please address:

a) Screening and extraction processes

To ensure a systematic and objective process of identifying relevant literature, please provide details on the pre-specified eligibility criteria for screening with reasons for exclusion, pre-defined data extraction form, number of researchers for independent searching, and how to handle any discrepancies.

**Response**

We have now expanded the methods section on bibliometric analyses to give an account of identifying relevant literature, eligibility criteria for inclusion of studies and data extraction process. However, since this is not a systematic review, we did not follow PRISMA recommendations. These processes are also outlined in several publications in PloSOne:

1) Zongyi Y, Dongying C, Baifeng L. Global regulatory T-cell research from 2000 to 2015: a bibliometric analysis. PLoS One. 2016 Sep 9;11(9):e0162099.
2) Shen S, Cheng C, Yang J, Yang S. Visualized analysis of developing trends and hot topics in natural disaster research. PLoS one. 2018 Jan 19;13(1):e0191250.
3) Waqas A, Salminen J, Jung SG, Almerekhi H, Jansen BJ. Mapping online hate: A scientometric analysis on research trends and hotspots in research on online hate. PloS one. 2019;14(9).

In addition to changes mentioned above, the ***Bibliometric analysis*** in methods now includes following information:

*For bibliometric analyses, the data set (n=260) was screened to include original research publications published from the South Asian birth cohorts. Only original studies published from birth cohorts conducted in Pakistan, India, Bangladesh, Afghanistan, Nepal, Bhutan and Maldives were included. Out of the 260 full texts, 38 studies were excluded for reasons including regions other than South Asia (n= 21), other study designs (n= 11), duplicate texts (n= 4) and literature reviews or systematic reviews (n= 2). Then, data pertaining to characteristics of these studies were manually extracted from abstracts and entered in Microsoft Excel sheets.*

**Comment 6**

Uniqueness of Pakistan

Please elaborate the uniqueness of Pakistan in South Asian settings, including similarities and differences in terms of historical context, social, economic and political development, and hence their exposures and confounding structures.

**Response**

A new paragraph on Pakistan’s unique sociocultural environment has been added in the revised manuscript:

*We also offer recommendations on establishing a new birth cohort in Pakistan; a country that boasts a unique sociocultural setting in the region. Pakistan, officially the Islamic Republic of Pakistan, is a predominantly Muslim country. With a population exceeding 210 million people, it is the world’s sixth most populous country (Pakistan Bureau of Statistics, 2017). It boasts a rich cultural and social diversity representing minorities from Hinduism, Christianity, Sikhism and Buddhism. Major ethnic groups include the Punjabis, Sindhis, Pashtuns and Baluchis as well as many other minority groups. Pakistan also houses one of the largest Afghan refugee population. In addition to its ethnic diversity, the nation of Pakistan is also rapidly urbanizing- and has long suffered from political instability, wars and terrorism in the region. Pakistan also boasts a population growth rate of 2.40%- where children and adolescents make up over 35% of the population [21]. According to the World Health Organization, Pakistan also performs poorly on several socioeconomic and health indicators. Around 21% of the Pakistani population lives below the poverty line with poor access to healthcare. It also has one of the highest indicators of maternal and child mortality [21]. In 2018, Pakistan had a neonatal mortality rate of 42 (per 1000 live births), under five mortality rate of 69.3 and maternal mortality ratio of 140 per 100,000 live births [21].*

**Reviewer comments**

Reviewer Comment #1: To be considered a systematic map the authors need to provide:
- a rationale for searching only one database (minimum is usually two)
- to include the full search string for this search in a database
- full eligibility criteria
- a flow diagram (check PRISMA).

**Response**

Dear Sir or Madam, thank you so much for such valuable feedback on the manuscript. Based on your feedback, I believe the major change in the revised manuscript is its shift from a systematic mapping study design to a scientometric and bibliometric study design. These methods are very common in library sciences and knowledge mapping fields but a rare occurrence in healthcare sciences. PloSOne has recently published several studies in this domain as noted above. Therefore, keeping in mind the study design of scientomric analyses, we have restructured the manuscript as detailed above. We have given a rationale for choosing only one database and the eligibility criteria. We have also given details of studies excluded in methods section with reasons. However, we did not provide a PRISMA flowchart which would have been more relevant for systematic or scoping reviews.

**Comment 2**

The systematic map findings are quite dense and hard to follow. It is not possible to ascertain from the methods how the studies were coded lead to that analysis. They also veer from purely descriptive to reporting the potential contribution of studies to understand the burden of disease in the region. The latter appear to 'slip into the findings' here and there, leading the reader to make inferences, on studies which have not been critically appraised.

**Response**

We have removed any points in this section that would better fit in discussion section. Moreover, we have also deleted sentences related to quality appraisals of studies that was not an objective of this study. To make the processes of data abstraction clearer, we have divided the methods and results section into two sections as noted above.

To avoid any cognitive leaps from purely descriptive to reporting the potential contribution of studies. For instance, the following paragraph in results section has been deleted:

*Co-citation analyses of the limited research contributions from birth cohort studies in South Asia reveal that these are clustered into a few specific clusters pertaining to physical health. And in contrast to the British cohorts, there has been negligible contributions in the area of psychological health of mothers and children. Moreover, the recent holistic drive in global health in delineating the psychoscocial determinants of diseases- thus, defining physical and psychiatric morbidities using both the biological and psychosocial approaches, is unseen in the region.*

**Comment 3**

The review authors need to decide whether this is a literature review, systematic map or systematic map, and make appropriate revisions accordingly before it can be sufficiently peer reviewed. The work will the have greater scientific value and contribution to make to the field.

**Response**

We have now removed any mention of systematic mapping from the manuscript. The design of the study has now been correctly defined as scientometric analyses.
