# Supplementary material for: A scientometric analysis of birth cohorts in South Asia: Way forward for Pakistan
Source: PLoS One. 2020 Jul 9;15(7):e0235385. doi: 10.1371/journal.pone.0235385 (PMC7347181; doi:10.1371/journal.pone.0235385)
Supplement: Attachment — Submitted filename: PONE-D-19-24798 review.docx [file pone.0235385.s003.docx]

**General comments**

Ahmed Waqas and colleagues conducted a study on bibliographic records retrieved from the Web of Science to assess systematically map of birth cohort studies from the South Asian region, examine the major research foci and landmark contributions and then recommend establishing new birth cohorts in Pakistan.

260 articles, published during through December, 2018, were retrieved from the Web of Science core databases based on search string: “((Birth-cohort) AND (Pakistan* OR India* OR Bangladesh* OR Afghanistan* OR Nepal* OR Bhutan* OR Maldives))”.

They found that India, Bangladesh, and Nepal published majority of original studies in birth cohorts. Three major birth cohorts were the prospective and multi-country MAL-ED birth cohort, the Pakistan Early Childhood Development Scale Up Trial, and a retrospective Maternal and infant nutrition intervention cohort.

Although reviewers’ comments were carefully responded, there are some issues needed to be clarified.

**Specific comments:**

1. The search string was too strict. Only term “birth-cohort” was used to search on the Web of Science. When other terms (e.g. “birth cohort” or “birth cohorts”) were added into the search string, the number of articles was ~ three-fold increase (277 vs 882 - see the figure). This leads to a concern about the quality of study results.

2. There is not much insight in the topic and the results of this study. Personally, I think if authors check their search string and correct their data, it is suitable for publishing in a local journal.
