# Supplementary material for: A scientometric analysis of birth cohorts in South Asia: Way forward for Pakistan
Source: PLoS One. 2020 Jul 9;15(7):e0235385. doi: 10.1371/journal.pone.0235385 (PMC7347181; doi:10.1371/journal.pone.0235385)
Supplement: Attachment — Submitted filename: Response to reviewers final.docx [file pone.0235385.s004.docx]

**Reviewer #2: General comments**

Ahmed Waqas and colleagues conducted a study on bibliographic records retrieved from the Web of Science to assess systematically map of birth cohort studies from the South Asian region, examine the major research foci and landmark contributions, and then recommend establishing new birth cohorts in Pakistan.

260 articles, published during through December, 2018, were retrieved from the Web of Science core databases based on search string: “((Birth-cohort) AND (Pakistan* OR India* OR Bangladesh* OR Afghanistan* OR Nepal* OR Bhutan* OR Maldives))”.

They found that India, Bangladesh, and Nepal published majority of original studies in birth cohorts. Three major birth cohorts were the prospective and multi-country MAL-ED birth cohort, the Pakistan Early Childhood Development Scale Up Trial, and a retrospective Maternal and infant nutrition intervention cohort.

Although reviewers’ comments were carefully responded, there are some issues needed to be clarified.

**Response**

On behalf of our team, I would like to thank the worthy reviewer for their excellent feedback. We had indeed tried our best to carefully respond to and revise our manuscript in line with reviewer’s suggestions in previous revisions. This had significantly raised the status of the manuscript.

Please, see point-by-point responses to reviewer comments below.

**Comment 1**

1. The search string was too strict. Only term “birth-cohort” was used to search on the Web of Science. When other terms (e.g. “birth cohort” or “birth cohorts”) were added into the search string, the number of articles was ~ three-fold increase (277 vs 882 - see Figure 1). This leads to a concern about the results of this study.

**Response**

- While we appreciate the reviewer’s comment, we had tried our best to conform to basic principles of scientometric research. Database search of Web of Science using the search term *(birth-cohort)* was carefully planned. This is because birth cohort designs are almost always used as a phrase in titles, abstracts and full texts of manuscripts. Our search terms (birth-cohort) would also produce the same results if we used the search terms suggested by the reviewer, such as “birth cohort”, because both the hyphen and quotation marks signify searching a phrase and can be used interchangeably in scientometric research.
- The worthy reviewer has rightfully reported that use of search terms in their screenshot (Figure 1) (birth-cohort OR birth cohort), provides 882 results. I think this may be because the second search term *birth cohort* has not been enclosed in quotation marks, and thus, was searched as two independent terms rather than as a phrase. while running this search strategy, it is important to know that a phrase search should always have two terms joined either by a hyphen or placed in quotation marks. And if you run a search for two separate terms (birth cohort), most of the databases just like Web of Science would consider birth as a separate term and cohort as a separate term, with their appearance in title of different studies disjointed. Let me please demonstrate this in Figure 2, where I use the search terms *(birth cohort)* (Figure 2). You could note here that the top 4 search results are not birth cohorts by any measure, and that this search would yield irrelevant research studies.
- Nevertheless, as a precaution and acting upon the reviewer’s advice, we ran another candidate of search terms, TS= (birth AND cohort) AND CU= (Pakistan* OR India* OR Bangladesh* OR Afghanistan* OR Nepal* OR Bhutan* OR Maldives). This yielded a total of 982 search results, which were carefully screened. After screening, we only found 214 relevant studies and rest of the articles were irrelevant. These 214 terms did not identify any new South Asian cohorts compared with our initial search that identified 222 publications. Following is a breakdown of reasons for exclusion of studies:

| **Reason** | **Number** |
| --- | --- |
| wrong study design | 539 |
| Trial data | 74 |
| retrospective | 44 |
| wrong population | 32 |
| cross-sectional | 25 |
| wrong study duration | 14 |
| RCT data | 12 |
| wrong publication type | 5 |
| Not from South Asia | 5 |
| Systematic Reviews | 3 |
| wrong outcome | 1 |
| duplicate | 1 |

- Use of a tighter search strategy is common in scientometric literature, to facilitate analysis of relevant studies. However, the importance of a more complex search strategy cannot be denied, and is particularly important when conducting systematic reviews to address a focused research question. In contrast to systematic reviews, scientometric analyses have a very different approach. The focus here is analysis of bibliographic data and studying relationship between two or more publications by leveraging the theory of co-citation analysis. The focus here, is not the publications/studies themselves or looking at what the authors did or the quality of study design, but rather getting a *feel* for the *trends* of research by analyzing the bibliographic data of these studies (cited) as well as the references they are cited in (citer). These results are then analyzed using network analysis and clustering algorithms rather than manual data extraction as we do in systematic reviews.
- The issue of relevant vs irrelevant search results and use of singular keywords has been debated previously in several publications. Emphasis is placed on finding relevant articles here because while doing scientometric analysis, data files cannot be manually edited or screened according to an eligibility criterion, as we do in systematic reviews. If done manually, it would corrupt the data files. To avoid the issue of irrelevant publications (which is the result of a poor database search), it is important to devise a good strategy, that is specific as well as inclusive of the domain under investigation. And that is the reason I had searched using a simple search strategy that I had devised.

Use of single terms thus, has been emphasized by several publications, for instance:

a) Term: “aristolochic acid”:

Citation: Zhou Q, Pei J, Poon J, Lau AY, Zhang L, Wang Y, Liu C, Huang L. Worldwide research trends on aristolochic acids (1957–2017): Suggestions for researchers. PloS one. 2019;14(5).

Comment: They specifically mention: "In the initial step of the bibliometric literature analysis, we used “aristolochic acid” as the search term in the WoS database and retrieved 1,603 documents. As part of the search, we performed several queries in succession, such as using “aristolochic acids” or “aristolochic acids and derivatives” as the search term. Finally, we used “aristolochic acid” as the retrieval term because it led to almost every relevant search result."

b) Use term "systems biology" to study trends in this domain:

Citation: Zou Y, Laubichler MD. From systems to biology: A computational analysis of the research articles on systems biology from 1992 to 2013. PloS one. 2018;13(7).

c) Term: ‘Treg*’ and ‘regulatory T cell*’

Citation: Zongyi Y, Dongying C, Baifeng L. Global regulatory T-cell research from 2000 to 2015: a bibliometric analysis. PLoS One. 2016;11(9).

d) Term: "digital medic*":

Citation: Fang Y. Visualizing the structure and the evolving of digital medicine: a scientometrics review. Scientometrics. 2015 Oct 1;105(1):5-21.

e) Term: “regenerative medicine”:

Citation: Chen C, Hu Z, Liu S, Tseng H. Emerging trends in regenerative medicine: a scientometric analysis in CiteSpace. Expert opinion on biological therapy. 2012 May 1;12(5):593-608.

- In my previous experience there have been instances, when I used a really broad search strategy and did end up with many irrelevant articles in a scientometric analysis. Even then, we do not screen the data manually, we do rather recognize the irrelevant collection of articles during analysis and exclude them. For instance, please see Figure 7 in:

Waqas A, Salminen J, Jung SG, Almerekhi H, Jansen BJ. Mapping online hate: A scientometric analysis on research trends and hotspots in research on online hate. PloS one. 2019;14(9).

You would note that *qsar analysis* is the very first term in a paper on online hate. Now what is this Quantitative structure-activity relationship (QSAR) technique from pharmacogenetics doing here? This was because we used several keywords including toxicity which brought a whole cluster of QSAR analysis in a paper on cyberbullying.

- The basic principle of a scientometric analysis as in our case is that we analyze a subset of top cited research studies and the papers these are cited in. Having a narrower search strategy, therefore, is not a problem, because we analyse not only the articles retrieved from the database but also the references in which these articles are cited in. So, whatever analysis we do is based on thousands of article title/references, which help us to analyse trends of research. And even if I had 1000s of results, it would still not matter, because I only take a pool of top 50 cited articles and their citee references to analyse.

**Revision made to paper in response to this comment**:

- The above reasoning has now been summarized and added in our manuscript for interested readers. In addition, we also provide a paragraph on scientometric analyses and their significance.

a) Please, see revised section on academic search
b) Please, see a new section on Scientometric analysis: An introduction.

Figure 1: Worthy reviewer’s WOS search

Figure 2: Using birth cohort as separate terms TS=(birth cohort)*

*Studies from 2-4 are not birth cohorts.

**Comment 2**

There is not much insight in the topic and the results of this study. Personally, I think if authors check their search string and correct their data, it is suitable for publishing in a local journal.

**Response**

Utilizing the scientometric techniques, we have tried our best to provide insights that can be utilized for stakeholders to provide an influx of personnel, infrastructure and funding resources where it is most required. We also outline the major themes of research that have previously repeatedly been searched upon such as disorders such as diabetes, metabolic syndrome and infections.

The study provides several insights regarding birth cohort research being done in the region. And using our analyses, we were able to draw several inferences and give recommendations on how to prioritize birth cohort research in Pakistan and neighboring countries. We also found several gaps that should be addressed if we are to provide impact on par with the British or European birth cohorts. The recommendations given in the previous version of the manuscript are:

- *Maternal, reproductive and infant health and development*: Collect detailed information on factors affecting maternal and infant health and development, to identify approaches to improve these key outcomes for future health and productivity and inform intervention studies.
- *Non-communicable diseases:* Identify early-life causes of, and predictors for, type 2 diabetes, cardiovascular disease and common mental disorder in Pakistan and inform novel interventions to prevent or address these.
- *Health systems:* Allow systems level analysis of data and act as a stimulus and test bed for strengthening health systems building blocks, including staffing, information and service delivery.
- *Poverty:* Poverty is a key determinant of health, and poor health of a family member can lead to catastrophic poverty[73]. It should allow in-depth study of:

(i) Interplay between health and poverty, and the extent to which there is a vicious cycle between the two across generations

(ii) The extent to which poverty-reduction schemes, such as conditional cash transfer, both reduce poverty and improve health and free access to health, on long-term health goals.

(iii) Understanding the barriers to and facilitators of implementing poverty-reduction schemes by national and local governments, and access, uptake and continued participation in the schemes by different families.

(iv) Understanding the relationship of poverty and poverty-reduction schemes to resilience and the ability to make health change and subsequent impacts on physiology, psychology and health.

- *Environment:* Pakistan is projected to be one of the most-affected country due to effects of climate change [74–76]. Over-crowding and water shortage are likely to be major challenges in the coming decade. It should help understand the impact of environment on health and solutions to reduce the impact, such as access to clean energy and water.
- *Genetics:* Pakistan has one of the highest rates of consanguineous marriages [77]. The high prevalence of human knockouts in the population is potentially highly informative about human health and development of innovative treatments.
- *Interdisciplinary collaborations:* Allow interdisciplinary collaborations within Pakistani and international academic communities. Develop the biobank and measurement variables keeping cutting edge technologies such as new techniques in genomics, nanotechnology and data sciences.
- Major research output in South Asia has been limited to the context of two countries: India and Bangladesh; and a limited aforementioned research foci. Therefore, there is a need to establish a birth cohort that answers research questions in Pakistani context, builds on the strengths and weaknesses of previous cohorts, and further augments their scope by taking mental health, social development and psychosocial environment in focus. This would also address the need of establishment of biological banks storing biological specimens. Thus, aptly putting psychosocial into cutting edge biological research.
