# Supplementary material for: A scientometric analysis of birth cohorts in South Asia: Way forward for Pakistan
Source: PLoS One. 2020 Jul 9;15(7):e0235385. doi: 10.1371/journal.pone.0235385 (PMC7347181; doi:10.1371/journal.pone.0235385)
Supplement: Attachment — Submitted filename: Response to reviewers.docx [file pone.0235385.s005.docx]

Dr. Man Ki Kwok

Academic Editor

PloSOne

Dear Dr. Man Ki Kwok,

On behalf of our team, we are grateful to you for a timely review and an excellent feedback on our manuscript. We have now revised our manuscript in line with the reviewer’s feedback, which has improved the quality of our manuscript.

Please, find point by point responses to reviewer’s comments below.

Best wishes,

Dr. Ahmed Waqas

Corresponding author

**Comments**

**Reviewer #2**

**Comment 1**

Figure 1 needs to show name of each country in the map. It is better to use shades of a color to reflect the numbers of original publications rather than use different colors.

**Response**

We have now provided detailed legends to the map chart in the revised manuscript. The detailed legend includes name of the country and corresponding number of publications.

**Comment 2**

Figure 2 & 3 do not have x-axis and y-axis labels. The figures also need to show values in bars and lines.

**Response**

X and y-axes in both the figures have now been labelled.

**Comment 3**

Figure 4, 5, 6, & 7 are quite dense and hard to follow. Authors should provide more description in figures to make reader understand what information represented (e.g. edges, nodes, clusters, colors) and the key points they want to show.

**Response**

We have now added detailed legends to the figures, with an example to interpret the clusters and their labels.

**Comment 4**

Line 249 mentions three main themes but the text lists 4 themes (a, b, c, d). It will be easier to follow if these themes are demonstrated in Figure 5.

**Response**

Line 249 has been edited to reflect four themes instead of three.

**Comment 5**

Some labels in Figure 7 are overlapped.

**Response**

The overlapped labels have now been corrected and an edited figure has been provided replacing the old one.
