# Supplementary material for: A scientometric analysis of birth cohorts in South Asia: Way forward for Pakistan
Source: PLoS One. 2020 Jul 9;15(7):e0235385. doi: 10.1371/journal.pone.0235385 (PMC7347181; doi:10.1371/journal.pone.0235385)
Supplement: Attachment — Submitted filename: Response to reviewers.docx [file pone.0235385.s006.docx]

Dr. Man Ki Kwok

Academic Editor

PloSOne

Dear Dr. Man Ki Kwok,

On behalf of our team, we are grateful to you for a timely review and an excellent feedback on our manuscript. We have now revised our manuscript in line with the reviewer’s feedback, which has improved the quality of our manuscript.

Please, find point by point responses to reviewer’s comments below.

Best wishes,

Dr. Ahmed Waqas

Corresponding author

**Reviewer #2**

**Comments**

**Figure 1:** Please check the colors of Bhutan and Afghanistan. It is fine to use different colors to demonstrate countries. However, a specific color pallet or different shades of one color are better to reflect the difference in numbers of publications among the countries.

**Response:** We have now updated the figure with suggested revisions. A specific color pallet has now been to reflect differences I number of publications among countries rather than individual countries.

**Figure 2:** Please be specific in Y-axis (i.e. Number of Publications). Show the number in each bar.

**Response:** Y-axis has been retitled to “Number of publications”. Numbers in each bar have now been shown. However, after consultation, the bar chart was converted to line graph for better visualization of findings.

**Figure 3:** It is necessary to distinguish between rate/ frequency and number of citations. The text mentioned “number of citations” and the Y-axis shows “Frequency of citations” while the label indicates “The citation rates of citations”.

**Response:**

We have now revised these. The text in the title, axis and text has been standardized to number of citations. These read as:

**“Figure 3:** Year-wise number of citations accrued by publications on birth cohorts published from South Asia

**Legend:** Year-wise number of citations achieved by papers, pertaining to birth cohorts published in South Asia

**Text:** The number of citations received by the publications rose from < 10 to over 750 from the year 2000 to 2018.”

**Figure 6,7,8,9:** In each figure, please describe the information that the edge, node, clusters, and color indicate.

**Response:**

For these figures, we have now provided very detailed legends, which should be published alongside each figure. Noting these sentences within the figures themselves would clutter information. These detailed legends read as:

**“Figure 6:** Research Foci from 2005-2010

This figure depicts major themes and foci of research output stemming from different birth cohorts established in South Asian Region, from 2005 to 2010. The shaded regions represent different clusters of research, labelled using indexed keywords. For instance, cluster # 1 in birth cohort research from 2005 to 2010 has been labelled as *leg height ratio and thickness*. Several nodes depicting number of citations as tree rings are shown to represent important entities. Purple coloured nodes represent landmark works that connect two clusters. Edges presenting relationship between two nodes are presented as lines. Different clusters are depicted with different colours.

**Figure 7:** Research Foci from 2011-2015a

This figure depicts major themes and foci of research output stemming from different birth cohorts established in South Asian Region, from 2011 to 2015. The shaded regions represent different clusters of research, labelled using indexed keywords. For instance, cluster # 0 is labelled as *lipid levels*. Several nodes depicting number of citations as tree rings are shown to represent important entities. Purple coloured nodes represent landmark works that connect two clusters. Edges presenting relationship between two nodes are presented as lines. Different clusters are depicted with different colours.

**Figure 8:** Research Foci from 2011-2015b

This figure depicts major themes and foci of research output stemming from different birth cohorts established in South Asian Region, from 2011 to 2015. The shaded regions represent different clusters of research, labelled using indexed keywords. For instance, cluster # 4 represents research on time series analysis. Several nodes depicting number of citations as tree rings are shown to represent important entities. Purple coloured nodes represent landmark works that connect two clusters. Edges presenting relationship between two nodes are presented as lines. Different clusters are depicted with different colours.

**Figure 9:** Research Foci from 2016-2018

This figure depicts major themes and foci of research output stemming from different birth cohorts established in South Asian Region, from 2015 to 2018. The shaded regions represent different clusters of research, labelled using indexed keywords. For instance, cluster # 13 represents research on *environmental enteropathy*. Several nodes depicting number of citations as tree rings are shown to represent important entities. Purple coloured nodes represent landmark works that connect two clusters. Edges presenting relationship between two nodes are presented as lines. Different clusters are depicted with different colours.”
